# New Symptom in Pneumonia

**Published:** 1846-05

**Authors:** 


*New Symptom in Pneumonia.*

In examining the sputa in a patient affected with pneumonia, M. Remak of Berlin, was surprised to find fibrous filaments in the matter ordinarily expectorated, very dense, and ramified nearly like the bronchial subdivisions. At first he regarded them as accidental, but his attention being fixed on the appearance, he soon came to the conclusion that these "ramified bronchial concretions," as he terms them, were a constant ingredient of the sputa in pneumonia. He witnessed their appearance in nearly fifty cases, and now considers them as among the most constant and certain indications of the disease; they are never entirely absent, although some variety may be observed in the period at which they appeared, their duration, quantity, &c. To discover these, it is often only necessary to pour the expectorated matter into a vessel of water; those only of some length and thickness, and which are not involved in mucus, can be seen, but if they are slender and weak, or involved in blood, mucus, or other matter, the vessel must be flat and of a dark colour, as a blackened piece of glass. It is sometimes necessary to leave the expectorated matter a long time in the water, and even then without being accustomed to their appearance, they may escape superficial search. In some of his patients they have been found nearly three inches long and a line thick; the trunk, as it may be called, of one of these is generally more slender than the sum of its branches, which latter become filiform at their extremities. It is not uncommon to find a slight enlargement where they bifurcate, corresponding perhaps to the enlargements in the bronchi at their ramifications. Sometimes they are flattened—sometimes nodulous, the nodules occasionally enclosing bubbles of air that cause them to float on the water, but when it escapes, the matter at once sinks to the bottom. Microscopic examination of these concretions show them to be formed of extremely delicate filaments, parallel and adherent to each other, and in the direction of their length; in most cases these filamants enclose a great number of granular cells so analogous to those of pus, from their firmness and appearance, that one can hardly avoid considering them as of a fibrous nature, but he will soon discover his error. By plunging the filaments into acetic acid they become perfectly transparent, and there remain in the liquid a great number of granulations, but they have not the elongated form peculiar to those of true fibrous tissues. He never observed in them, even when immersed in water, those molecular motions noticed in other granules, particularly those of the bronchial mucus, and they are much smaller than those of pus. Those concretions are mostly observed to make their appearance from the eighth to the seventh day of pneumonia; they rarely are absent on the fourth or fifth day, and not in a few cases are they seen on the second day or after the seventh; this limit, however, appears only in those cases where appropriate treatment was had recourse to from the beginning of the disease. The expectoration in pneumonia is of three kinds—the first, a filamentous mucus, greyish, or oftener tinged with very viscous blood adhering to the vessel into which it falls; at a later period it is a whitish mass, dense, granular, whose white filaments are slender, of the consistence of mucus, and sink rapidly to the bottom of the vessel; in the last stage the expectorated matter is soft, conglobate, of a whitish or yellowish colour, puriform, and without filaments; the concretions are recognized in the two first forms of sputa, but never in the third. When met with in the first form they are of remarkable tenuity, like fine thread, and with very few branches, but are very firm and much ramified in the second stage. When these concretions are examined in water, it is easy to confound them with the white mucous filaments often met with; we easily discover the difference by placing the expectorated matter on a bit of glass, the mucous filaments can be readily expanded into a fine membrane with the point of a needle, while the consistence of the concretions will not permit this transformation, and besides, the branchings, which are never found in the mucous filaments, will distinguish the one from the other at once. It is important to consider the signs afforded by auscultation and percussion which accompany the appearances of those concretions; they mostly appear at the period when, on the one hand, the *rale crepitant* is most marked, and when, on the other hand, the augmenting loudness indicates the partial impermeability of the lung. In general we do not find these concretions well organized until the *rale crepitant* has almost entirely given place to the loud bronchial *souffle*, and when the dulness of the part denotes the complete hepatization of the lung. As to the nature of these concretions it is impossible to consider them otherwise than as a fibrinous exudation formed by the granular cells: they must be of a fibrous nature, for although the amorphous mucus can sometimes form well-marked filaments, yet these never possess the firmness and consistency which peculiarly belongs to the bronchial concretions; chemistry, too, confirms this opinion; the concretions are dissolved by caustic potash at the temperature of boiling water—the addition of acetic acid renders the solution turbid, which an excess again dissolves; carbonate of ammonia throws down a very fine flocculent precipitate; it should not be concluded from this that in their elementary composition they are analogous to the fibrine of the blood —a number of chemical analyses are wanting still to determine such an analogy, both with respect to them and to other fibrous exudations found on the surfaces of mucous and serous membranes. As regards the practical relations of those concretions, it has been observed by M. R. that perfect recovery is more certain and rapid when they have come on late and in proportion as they are abundant and persistent. In the natural course of pneumonia, in robust persons, the concretions are found in the first viscid mucus expectorated; they augment in quantity and consistence to the fifth day; they then gradually diminish, and are replaced by small white friable masses still of a cylindrical form, but without ramifications, and which under the microscope display the fibrous structure, and the presence of mucous corpuscles in granules. Patients seldom come to the hospital until one or perhaps several days have elapsed from the commencement of the attack, and the change of place is not, perhaps, without its influence on the functions of the lungs, and may check the natural course of the disease; it is not unfrequent, then, not to find the concretions in the sputa when the patient applies for admission, but appear some days, after the adoption of the antiphlogistic treatment. In other cases where the concretions appear at a later period, the irregularity may be traced to something wrong in the patient's constitution, and particularly in the existence of pulmonary tubercles. Among fifty cases there were but four or five in which the disappearance of some morbid phenomena had preceded the appearance of the concretions; in most cases the amelioration has been subsequent. M. Schönlein, who became acquainted with these appearances in the sputa through M. Remak, considers them of great importance as a new symptom of pneumonia; he thinks the appearance of a large quantity of the concretions in the expectorated matter indicates that the time for general bloodletting has passed, but that still recourse is to be had to topical bleeding, mercurial frictions, or the external use of iodine, diaphoretic and cooling drinks, as the infusion of digitalis with nitrate of potash, and finally to resolutives, calomel, blisters, &c. M. Remak thinks that as the elasticity of the lungs disappear when hepatized, it will often, perhaps be useful to follow rapidly the antiphlogistic treatment by expectorants, as the golden sulphuret of antimony, or the polygala senega. M. Schönlein declares he has never seen a case of pneumonia in which there was not a corresponding sensibility of the skin, which he compares to that in the skin of the abdomen in certain cases of peritonitis—remarking, however, that in this latter case the sensibility was not always in proportion to the violence of the inflammation, and sometimes remained after this had been subdued.—*Dublin Med. Press, from Arch. Gen.*

